# Serum Calprotectin Is a Valid Biomarker in Distinction of Bacterial Urinary Tract Infection From Viral Respiratory Illness in Children Under 3 Years of Age

**DOI:** 10.3389/fped.2022.768260

**Published:** 2022-03-14

**Authors:** Mirta Lamot, Marijana Miler, Nora Nikolac Gabaj, Lovro Lamot, Milan Milošević, Miroslav Harjaček, Slaven Abdović

**Affiliations:** ^1^Division of Neonatology, Department of Gynecology and Obstetrics, Sestre Milosrdnice University Hospital Center, Zagreb, Croatia; ^2^University Department of Chemistry, Sestre Milosrdnice University Hospital Center, Zagreb, Croatia; ^3^Faculty of Pharmacy and Biochemistry, University of Zagreb, Zagreb, Croatia; ^4^Department of Pediatrics, Sestre Milosrdnice University Hospital Center, Zagreb, Croatia; ^5^Department of Pediatrics, University of Zagreb School of Medicine, Zagreb, Croatia; ^6^Andrija Ṡtampar School of Public Health, University of Zagreb School of Medicine, Zagreb, Croatia; ^7^Division of Pediatric Nephrology, Department of Pediatrics, Children's Hospital Zagreb, Zagreb, Croatia

**Keywords:** calprotectin, urinary tract infection, bacterial infection, biomarker, pediatrics, respiratory viral diseases

## Abstract

**Background:**

Febrile illnesses in young children can be a major diagnostic challenge, despite the routine use of various laboratory markers. Recent advancements in the understanding of inflammatory processes have highlighted the role of calprotectin, a heterodimer consisting of S100A8 and S100A9 proteins, with many studies suggesting its clinical value as a biomarker of inflammation. This research aimed to evaluate the usefulness of serum calprotectin (sCal) as a biomarker of urinary tract infection (UTI), which was due to its high pooled prevalence and feasibility of urine culture as a diagnostic reference standard selected for a model of bacterial infection in children.

**Methods:**

Febrile children aged 0–36 months with suspected UTI based on positive urinalysis or viral respiratory tract infection were included. Children with significant bacteriuria in urine culture were labeled as cases (*n* = 58), while those with confirmed viral infection (*n* = 51), as well as those with suspected UTI but sterile urine culture who went on to develop symptoms consistent with viral respiratory infection (*n* = 7), were labeled as controls. sCal levels were determined by a commercial immunoassay. Conventional inflammation markers (C-reactive protein, procalcitonin, white blood cell count, absolute neutrophil count, and neutrophil percentage) were measured on the day of the clinical examination. Differences in measured inflammatory markers between cases and controls were analyzed with Mann-Whitney *U*-test. ROC analysis reported cut-off values with the best sensitivity and specificity to distinguish bacterial UTI from viral respiratory infection.

**Results:**

All analyzed inflammatory biomarkers, including sCal, were significantly higher in cases than in controls. Median concentration of sCal was 4.97 μg/mL (IQR 3.43–6.42) and 2.45 μg/mL (IQR 1.63–3.85) for cases and controls, respectively (*p* < 0.001). For identifying bacterial UTI, sensitivity and specificity of sCal were 77.6 and 69.0%, respectively, at an adjusted cut-off point of >3.24 μg/mL (AUC 80.2%).

**Conclusion:**

sCal could have substantial added value in the management of a child with fever and positive urinalysis and is a promising biomarker in distinction between bacterial UTI and viral respiratory causes of febrile illness in children under the age of 3 years.

## Introduction

A number of recent studies have shown a significant interest in comprehending the clinical diagnostic potential of monitoring calprotectin concentrations in blood and body fluids (1). Due to its ubiquity during infection and/or inflammation and stability at room temperature, it comes as no surprise that calprotectin has been by now isolated from feces, urine, saliva, cerebrospinal fluid, meconium, synovia, and serum/plasma (1, 2). Fecal or blood-based calprotectin seems to be an effective diagnostic and follow-up biomarker in inflammatory bowel disease (IBD), rheumatoid arthritis (RA), spondyloarthritis, juvenile idiopathic arthritis (JIA), ANCA associated vasculitis, systemic lupus erythematosus (SLE), and Kawasaki disease, where it is used as a predictor of disease relapse, response to treatment, and structural damage (3–6). Additionally, it has been shown that plasma calprotectin levels might be used to distinguish the bacterial from viral infection (7). In adults, calprotectin exhibited a great sensitivity and specificity in the discrimination between bacterial pneumonia and viral respiratory infections, while in preterm and term infants with culture proven or high probable sepsis, its sensitivity, as well as positive and negative predictive values, were higher than those reported for conventional infection markers (8–10).

Despite the routine use of various clinical and laboratory markers, febrile illnesses in children younger than 3 years of age can be a major diagnostic challenge for physicians caring for children (11). Although self-limiting viral respiratory infections are the principal cause of fever in this group, a considerable portion of children will develop a bacterial urinary tract infection (UTI) that requires antibiotic treatment. The most commonly used inflammatory markers such as white blood cell count (WBC), C-reactive protein (CRP), and procalcitonin (PCT) aid in identifying children at risk for bacterial infection, though their sensitivity and predictive ability are limited. Generally, both CRP and PCT perform better than WBC, while in the youngest children with serious bacterial infection PCT outperforms CRP in the very first hours from fever onset, but with much higher cost (11).

Calprotectin (referred to by various authors as L1, 27E10 antigen, CFA, MRP8/14, calgranulin A/B, and S100A8/S100A9) is a calcium binding protein named after its protective, antimicrobial properties (12, 13). It is expressed primarily in neutrophils and to a much lesser extent in monocytes, and its production is induced by the pro-inflammatory cytokines TNFα and IL1β *via* transcriptional factor C/EBPα (14, 15). The pathways which induce calprotectin expression and secretion during bacterial infection start with bacterial lipopolysaccharides (LPS) binding to a toll-like receptor 4 (TLR4) on phagocytes (16). On the other hand, calprotectin is designated as a damage associated molecular pattern protein (DAMP) which acts by binding to two pattern-recognition receptors, TLR4 and RAGE (receptor for advanced glycation end products), on innate immune cells, releasing numerous inflammatory mediators, including calprotectin. Hence, calprotectin acts in paracrine and autocrine manner to amplify acute immune response, and has a potential to indicate inflammation (16, 17).

To the best of our knowledge, no study has investigated the possible role of serum calprotectin (sCal) in differentiating viral from the bacterial cause of febrile illnesses in infants and children. This research aimed to evaluate the usefulness of sCal as a biomarker of bacterial UTI in children younger than 3 years of age, hypothesizing that sCal is significantly higher in children with proven bacterial UTI than in children with respiratory viral disease.

## Materials and Methods

### Participants and Procedures

This was a prospective study performed between October 2018 and February 2020 (before the first confirmed case of SARS-CoV-2 infection in Croatia) at the Department of Pediatrics in Sestre milosrdnice University Hospital Center, Zagreb, Croatia. The study involved children aged 0–36 months who were brought to Emergency Department due to a fever ≥38°C lasting <72 h and admitted to the inpatient or outpatient ward for further follow-up and treatment of suspected UTI (based on urinalysis) or respiratory tract infection. None of the participants had a history of chronic illness or ongoing antibiotic use at the time of visit.

Patients who turned out to have a UTI were regarded as cases, while those with proven viral respiratory infection, as well as those with sterile urine culture who went on to develop symptoms suggestive of viral respiratory infection, were regarded as controls. All of the participants had other source of bacterial infection excluded by negative pharyngeal swab, normal otoscopy finding, sterile blood culture, and/or negative chest radiography (if performed). The urine was collected as per the institution's protocol, with a sterile plastic bag attached to the perineum after thorough cleaning. Diagnosis of UTI was made with both positive urinalysis (leukocyte esterase greater than a trace amount and/or any positive nitrite detected by dipstick test) and significant bacteriuria in urine culture [100,000 colony forming units (CFU) of a single urinary tract pathogen per milliliter] (18–20). The diagnosis of viral respiratory infection was confirmed with rapid influenza or respiratory syncytial virus (RSV) immunoassays.

Every participant of the study underwent a medical history taking and clinical examination. Conventional inflammatory markers, such as CRP, PCT, WBC, absolute neutrophil count (ANC), and neutrophil percentage (N%), were measured on the day of the clinical examination with routine standards of the hospital laboratory.

WBCs were evaluated using Sysmex XN-1000 hematology analyzer (Sysmex, Kobe, Japan), CRP was measured by immunoturbidimetry method on Architect c8000, and PCT was determined by chemiluminescent microparticle immunoassay on Architect i2000sr, both Abbott, Abbott Park, IL, USA. The serum was frozen and preserved at −20°C until 69 samples were collected and analyzed for calprotectin at the same time. Two cycles were performed, which makes a total of 138 tested serum samples. SCal levels were determined using a commercial Quantum Blue^®^ sCal quantitative lateral flow assay on Quantum Blue reader (BÜHLMANN Laboratories AG, Schönenbuch, Switzerland). Test required 20 μL of serum, samples were diluted 1:10, and results were available after 20 min. Sensitivity of the test is <0.5 μg/mL.

### Ethical Considerations

All included patients provided a written informed consent to participation signed by their parent/guardian. The study protocol was approved by the institutional ethics committee and all procedures performed were following the ethical standards of the institutional ethics committee and the 1964 Helsinki declaration and its later amendments or comparable ethical standards.

### Statistical Analysis

Data distribution was checked with the Smirnov-Kolmogorov test. Where the assumption of normality was not upheld, the non-parametric tests were used. Continuous variables were expressed as the median and interquartile range (IQR). Data were analyzed for statistically significant differences using Mann-Whitney U test or Fisher exact test. The Kruskal Wallis test was used for determining whether the medians of sCal in 2 or more age sub-groups differ significantly, after which a Dwass-Steel-Critchlow-Fligner *post-hoc* test was performed to find out which of these groups differ from each other. Spearman correlation coefficient (*r*_*s*_) tested the significance of the correlation between sCal and values of standard inflammatory biomarkers. ROC analysis reported cut-off values with the best sensitivity and specificity to distinguish bacterial from viral infection. Univariate and multivariate binary logistic regression analysis were made for prediction of bacterial infection among febrile children. Statistical analysis was performed using SPSS for Windows (version 25; IBM Corporation, Chicago, IL, USA). ROC curve analysis was performed using MedCalc (version 19.1.7; MedCalc Software Ltd, Ostend, Belgium). Two-sided tests were used, with the level of statistical significance set at 0.05.

## Results

### Patient Selection and Demographics

**Figure 1:**
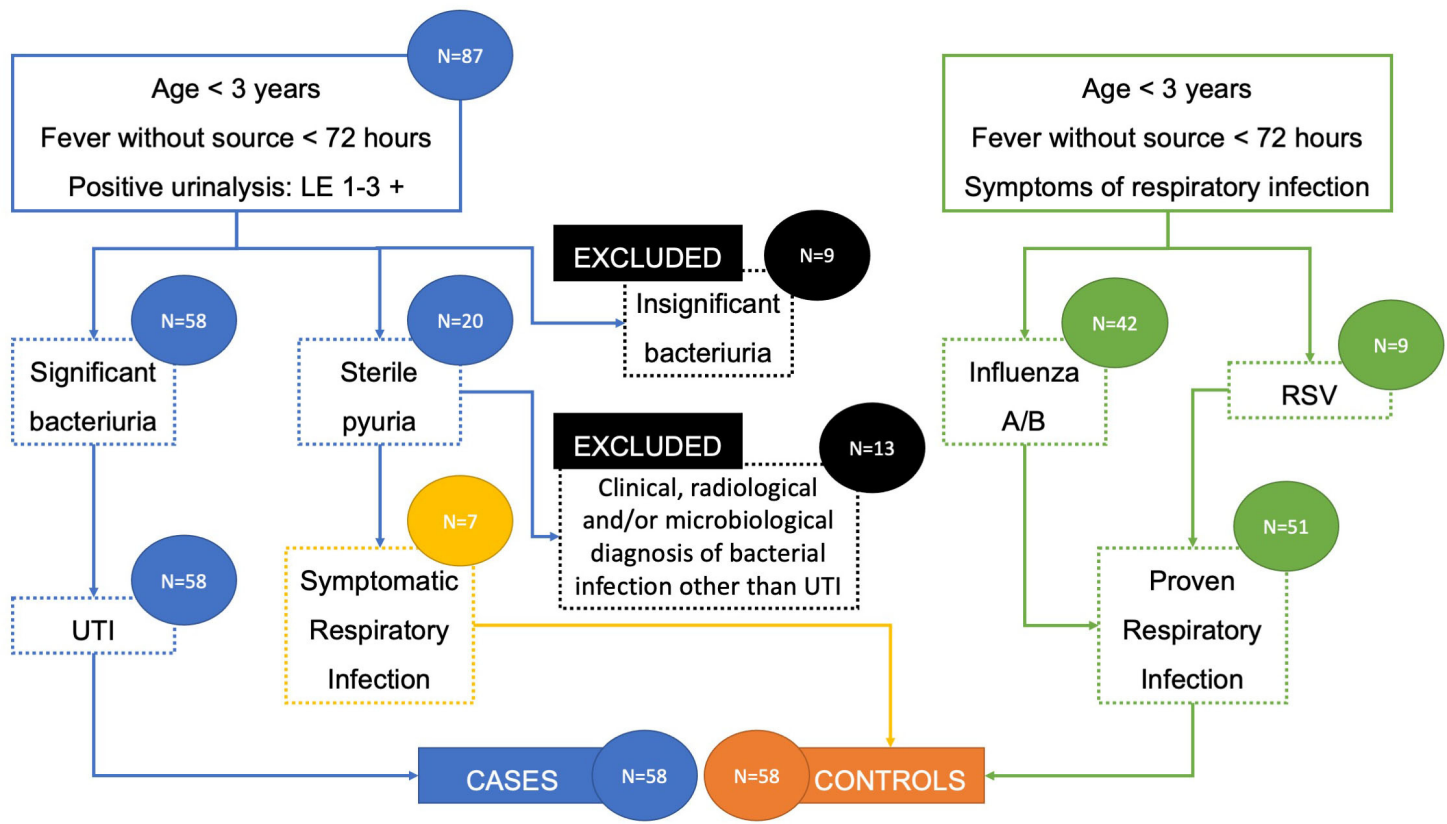
Patient selection flowchart.

A total of 138 patients were included in the study: 87 with suspected UTI and 51 with proven respiratory viral infection. Out of the patients with suspected UTI, 9 with insignificant bacteriuria (<100,000 CFU/mL) were immediately excluded, while 20 with sterile pyuria (no identified urinary tract pathogen) went for further assessment. Among these patients with sterile pyuria, 13 had clinical, radiological, and/or microbiological diagnosis of bacterial infection other than UTI and were excluded, while 7 with no such diagnosis of bacterial infection who further developed symptoms suggestive of viral respiratory infection were included in the control group. Therefore, the final number of participants was 116, divided into UTI cases group and respiratory viral infection control group, each consisting of 58 participants (Table 1). There were no significant differences in gender distribution between cases and controls, with 30 (51.7%) and 29 boys (50%), respectively. Figure 1 outlines the patient selection for the study enrollment.

**Table 1:** Microbiological isolates and their distribution in study participants.

**Participants**	**Diagnosis**	**Microbial etiology**	**No. of subjects**
Bacterial infection (*N* = 58)	UTI	*Escherichia coli*	55
		*Klebsiella pneumoniae*	2
		*Enterobacter* spp.	1
Controls (*N* = 58)	Confirmed respiratory viral infection	RSV	9
		Influenza A or B	42
	Symptomatic respiratory viral infection with sterile pyuria	Unknown	7

*UTI, urinary tract infection; RSV, respiratory syncytial virus*.

### Inflammatory Markers Levels

A statistically significant difference was found between participants and controls for all measured inflammatory biomarkers, including sCal. There was a strong positive correlation in cases between sCal and neutrophil count and percentage (*r*_*s*_ = 0.611 and *r*_*s*_ = 0.601, respectively, *P* < 0.001) and moderate between sCal and WBC and CRP (*r*_*s*_ = 0.491 and *r*_*s*_ = 0.446, respectively, *P* < 0.001). Table 2 shows differences in variables of interest between participants with bacterial infection and controls. There was no significant relationship between sCal and PCT (*P* = 0.081) in patients with proven bacterial infection (Table 3).

**Table 2:** Differences in variables of interest between cases with bacterial infection and respiratory viral controls.

	Bacterial UTI cases (*N* = 58^*^)	Respiratory viral controls (*N* = 58^*^)	***P-*value**
Age (months)	3.75 (2.0–6.13)	14.0 (5.0–21.25)	<0.001
Duration of fever (hours)	12.0 (5.75–24.5)	24.0 (12.0–48.0)	0.006
sCal (μg/mL)	4.97 (3.43–6.42)	2.45 (1.63–3.85)	<0.001
CRP (mg/L)	41.8 (21.1–104.38)	6.4 (1.68–16.28)	<0.001
PCT (ng/mL)	0.31 (0.11–2.05)	0.09 (0.06–0.2)	<0.001
WBC (10^9^/L)	17.95 (13.08–22.73)	7.8 (5.40–11.7)	<0.001
ANC (10^9^/L)	8.99 (6.35–12.01)	3.42 (2.02–5.19)	<0.001
N%	52.45 (45.28–61.43)	45.55 (33.73–55.9)	0.001

* *Number of participants with determined PCT levels were 45 and 36 for patients with bacterial infection and controls, respectively*.

*sCal, serum calprotectin; CRP, C-reactive protein; PCT, procalcitonin; WBC, white blood cell count; ANC, absolute neutrophil count; N%, neutrophil percentage*.

*For continuous variables, medians and interquartile ranges are shown*.

*For two-group comparison, significance was defined as P ≤ 0.05*.

**Table 3:** Correlation between sCal values and variables of interest.

	***r*** **_s_**	***N***	***P-*value**
Age (months)	0.416	58	0.001
Duration of fever (hours)	0.231	58	0.081
CRP (mg/L)	0.446	58	<0.001
PCT (ng/mL)	0.263	45	0.081
WBC (10^9^/L)	0.491	58	<0.001
ANC (10^9^/L)	0.611	58	<0.001
N%	0.601	58	<0.001

*CRP, C-reactive protein; PCT, procalcitonin; WBC, white blood cell count; ANC, absolute neutrophil count; N%, neutrophil percentage*.

*Strong positive correlation was defined as r_s_ (Spearman correlation coefficient) >0.5 and P ≤ 0.05*.

### Performance of Serum Calprotectin in Bacterial Infection

**Figure 2:**
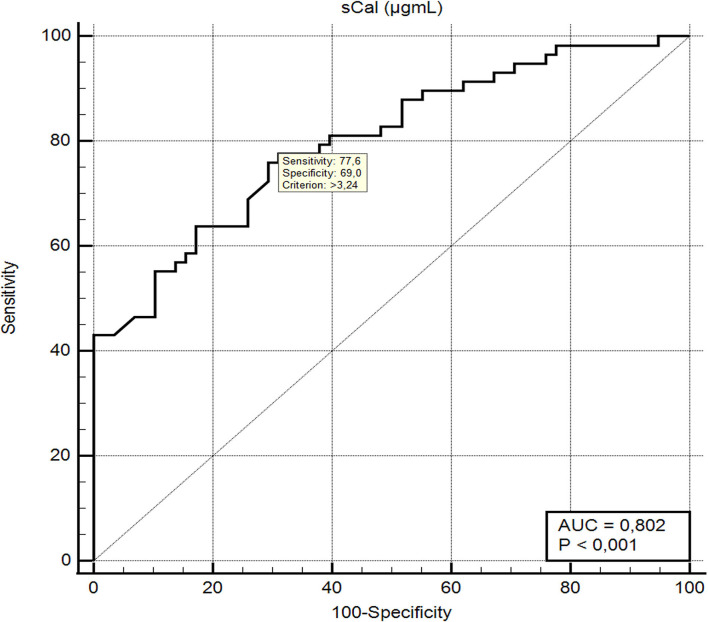
ROC curve and diagnostic performance of serum calprotectin in differentiation between bacterial UTI and respiratory viral infections.

ROC analysis (Figure 2) showed that sCal > 3.24 μg/mL has the best sensitivity (77.6%) and specificity (69.0%) according to the Youden index (*J* = 0.46; AUC = 80.2%, 95% confidence interval, 0.717–0.870). With the cut-off value set, a new binary variable (>3.24 or ≤3.24) was analyzed with the binary regression model. A significant prediction of bacterial infection was found for the value of sCal > 3.24 μg/mL with odds ratio 7.69 (95%CI: 3.35–17.65; *P* < 0.001). Table 4 summarizes AUC results and, at optimal cut-off value, sensitivity and specificity of all tested blood-based inflammatory biomarkers for the distinction between diagnosis of bacterial UTI and viral infection.

**Table 4:** Performance of all analyzed inflammatory biomarkers.

**Inflammatory marker**	**Cases/controls**	**AUC**	**Optimal cut-point**	**Sensitivity**	**Specificity**	**Accuracy**	**Youden index**
sCal	58/58	0.802	3.24 μg/mL	77.6	79.0	73.28	0.4655
CRP	58/58	0.8176	19.7 mg/L	75.86	77.59	76.72	0.5345
PCT	45/36	0.758	0.28 ng/mL	53.33	86.11	67.9	0.3944
WBC	58/58	0.8585	12.1 × 10^9^/L	84.48	77.59	81.03	0.6207
ANC	58/58	0.8433	6.18 × 10^9^L	79.3	84.48	81.9	0.6379
N%	58/58	0.6828	48.35%	63.79	67.24	65.52	0.3103

*sCal, serum calprotectin; CRP, C-reactive protein; PCT, procalcitonin; WBC, white blood cell count; ANC, absolute neutrophil count; N%, neutrophil percentage*.

### Serum Calprotectin Analysis Across Age Sub-groups of Participants With Bacterial Infection

Children with UTI were divided into 4 age groups: neonates (under 28 days of age), young infants (1–3 months), infants (4–12 months), and toddlers (13–36 months) (19, 21, 22). As shown in Tables 5, 6, sCal values in patients with bacterial infection were statistically significantly lower in neonates than infants (*W* = 4.140, *P* = 0.018).

**Table 5:** sCal median values across age sub-groups of participants with UTI.

**Age group**	***N***	**Median sCAL (IQR)**
Neonates	13	2.40 (1.65–5.10)
Young Infants	16	5.20 (3.63–5.93)
Infants	24	5.61 (3.84–8.70)
Toddlers	5	6.40 (4.19–8.76)

*Significant difference in median values of sCal between two or more age sub-groups was detected with Kruskal-Wallis non-parametric test (X^2^ = 11.1, P = 0.001)*.

*sCAL, serum calprotectin*.

*Neonates: under 28 days of age; Young infants: 1–3 months; Infants: 4–12 months; Toddlers: 13–36 months*.

**Table 6:** Pairwise comparisons of sCAL median values between age sub-groups of participants with UTI using the Dwass-Steel-Critchlow-Flinger (DSCF) *post-hoc* test.

**Pairwise comparison**		***W***	***P***
Neonates	Young Infants	3.071	0.131
**Neonates**	**Infants**	**4.140**	**0.018**
Neonates	Toddlers	3.137	0.118
Young Infants	Infants	1.445	0.737
Young Infants	Toddlers	2.103	0.446
Infants	Toddlers	0.653	0.967

*DSCF post-hoc test showed significant difference (P ≤ 0.05) in median values of sCal between neonates and infants (bold)*.

*W, Wilcoxon rank sum test statistic*.

*Neonates: under 28 days of age; Young infants: 1–3 months; Infants: 4–12 months; Toddlers: 13–36 months*.

### Binary Logistic Regression Analysis for Predicting Bacterial Infection Among Febrile Children

The results of univariate binary logistic regression analysis for association of all measured biomarkers (based on their AUC) with UTI in febrile children under 3 years of age are presented in hierarchical order in Table 7. WBC was the best predictor of UTI with *R*^2^ 0.32, while *R*^2^ of sCal and CRP were 0.24 and 0.21, respectively. In multivariate binary logistic regression analysis, when sCal was combined with WBC, its predictive value for UTI improved (*R*^2^ = 0.37, AUC 0.876). The model with CRP added to WBC and sCal was only slightly better (*R*^2^ = 0.40, AUC 0.882).

**Table 7:** The results (in hierarchical order) of univariate binary logistic regression analysis for association of all measured biomarkers (based on their AUC values) with UTI in febrile children under 3 years of age.

**Predictor**	**OR**	**95% CI**	***R*** **^2^ (%)**	***R*** **^2^ adj (%)**	**AUC**	***P***
WBC	1.2898	1.18–1.41	32.26	31.64	0.8585	≤0.001
ANC	1.4334	1.25–1.64	28.60	27.98	0.8433	≤0.001
sCal	1.9494	1.5–2.55	24.2	23.58	0.8017	≤0.001
CRP	1.0362	1.02–1.05	21.33	20.71	0.8176	≤0.001
PCT	1.9988	0.93–4.29	12.64	11.74	0.7580	0.076
N%	1.0522	1.02–1.08	8.45	7.83	0.6828	0.001

*sCal, serum calprotectin; CRP, C-reactive protein; PCT, procalcitonin; WBC, white blood cell count; ANC, absolute neutrophil count; N%, neutrophil percentage*.

## Discussion

The present study is the first to report the efficacy of sCal as a biomarker for the differentiation between bacterial UTI and viral respiratory infection in febrile children younger than 3 years of age. Moreover, it explored the performance of sCal relative to other inflammatory markers in children with bacterial UTI. A febrile bacterial UTI and viral respiratory infection were used as a model of bacterial and viral infection in children, respectively, due to its high pooled prevalence and feasibility of urine culture and nasopharyngeal aspirates as a diagnostic reference standard (23–25). Besides, clinical manifestation of febrile UTI in this age group often lack any signs and symptoms typical for children after 5 years of age, making UTI in a younger age group somewhat of a diagnostic dilemma (26).

According to AAP (American Academy of Pediatrics) and NICE (National Institute for Health and Care Excellence) guidelines, urinalysis is an important part of managing infants and young children presenting with unexplained fever of 38°C or higher (19, 20). Urine dipstick test is the recommended screening method for UTI in febrile children from 3 months to 3 years of age, and in age group 2 to 24 months has diagnostic sensitivity of 67–94% and specificity of 64–92% (19, 20). On top of that, readily available serum markers CRP and PCT are often used to prognosticate pyelonephritis in children with UTI. While CRP has been studied with conflicting results, more studies confirmed that PCT is a valuable indicator of acute renal involvement and the best predictor of permanent renal scarring in children under 2 years of age with first febrile UTI (27). Nevertheless, there is no compelling evidence to recommend the routine use of any of these tests in clinical practice (28). Moreover, as mentioned above, the reference standard for diagnosis of UTI is urine culture, which is time consuming and frequently leads to engagement of antibiotic treatment while pending for results, often not in line with the rules of rational pharmacotherapy (29). Finally, despite the increased availability of laboratory testing and development of clinical scores reachable online, such as UTIcalc, there is still no entirely accurate early predictor of UTI in children (30). Hence, establishing timely and reliable diagnosis of UTI in children presents an unmet clinical need.

The main presented finding in our study was that children with proven bacterial urinary tract infection had significantly higher sCal concentration than children with viral respiratory tract infection. This result is supported by Havelka et al. who recently reported that in adults with acute respiratory infections, plasma calprotectin levels were significantly higher in patients with bacterial pneumonia, mycoplasma pneumonia, and streptococcal tonsillitis compared to plasma calprotectin levels in patients with proven viral infections (10). Based on results of our multivariate binary logistic regression analysis, sCal has the best predictive value for UTI when combined with WBC (*R*^2^ = 0.37), while CRP has little added value (*R*^2^ = 0.40) in that combination. Moreover, the strongest correlation was observed between sCal values and neutrophils (absolute count and percentage), which can be explained by the fact that calprotectin is almost exclusively restricted to neutrophils and by now is already known as a marker for neutrophil mediated inflammation (2, 10, 15, 31).

In addition, sCal values correlated with CRP moderately, and there was no correlation with PCT, which reflects different aspects of the body's response to infection. Moreover, PCT in our study showed noticeably lower AUC for recognizing bacterial UTI than sCal (0.758 vs. 0.802), but this can be attributed to the selection bias, since PCT was routinely analyzed in only 62% of controls and 77.6% of cases (who all were probably more ill-appearing than the others) compared with 100% of study participants with defined sCal values. On the other hand, our results showed that other routinely measured inflammatory biomarkers (CRP, PCT, WBC, ANC, and N%) were significantly higher in cases of bacterial UTI than in controls with respiratory viral infection, with similar observed high performance AUC for identifying bacterial UTI.

We analyzed sCal values in four different physiologically meaningful age sub-groups of children under 3 years of age with bacterial UTI and recognized that neonates with UTI have lower sCal concentrations than infants with UTI. Unfortunately, the number of cases in each sub-group was too small to make relevant conclusions based on our findings. Nevertheless, this has raised an important point of how age influences sCal kinetics. Since currently the number of reliable studies on the matter is limited, a new study investigating reference interval of sCal in various age groups of children, especially neonates, is warranted.

Interestingly, Terrin et al. reported that in very low birth weight newborns with suspected sepsis, the diagnostic accuracy of sCal was greater (at cut-off value of 1.7 μg/mL sensitivity was 89% and specificity 96%) than the performance of CRP, WBC, and ANC (8). A few years later, Decembrino et al. published similar results in term infants with the same diagnosis: at cut off value of 2.2 μg/mL sCal was identified to distinguish between infants with and without sepsis with sensitivity and specificity of 62.5 and 69.7%, respectively, whereas CRP for a cut-off of 6.0 mg/L showed a sensitivity of 50% and specificity of 66.7% (9). The provided explanation was that CRP concentration increases rather slowly in the initial phase of inflammatory response to pathogens and many peripartum factors influence its kinetics. Considering there is no need for *de novo* synthesis and its rapid release from neutrophils, sCAL could be an earlier marker of bacterial infection, although this should be confirmed by a new study as well (32).

In our study, the participants were selected keenly and divided into two well-defined homogeneous groups according to the reference standard for the diagnosis of bacterial UTI or viral respiratory infection, which is the main strength of the study. Besides, comparison to other inflammatory markers was performed, providing data on the performance of sCal relative to the routinely used biomarkers.

The most important limitation of the study is a small number of participants from a single center. Thus, caution should be exercised, especially when interpreting findings of subgroups with small sample size. Additionally, instead of commonly recommended invasive urethral catheterization or bladder puncture, non-invasive bag technique was performed, thus the positive urine culture/significant bacteriuria was defined as presence of ≥10^5^ CFU of single urinary tract pathogen per milliliter of urine, while patients with lower bacterial counts or multiple pathogens were excluded from the study (18, 19). Nevertheless, this non-invasive collection technique could possibly influence the results of the study. Furthermore, the design of the study with only one, initial laboratory analysis on the first day of admission is insufficient for evaluating kinetics of inflammatory biomarkers, which makes the hypothesis that sCal as a biomarker of bacterial infection reaches its maximum level in blood before CRP and other routinely used inflammatory biomarkers only speculative. Also, there were some significant differences in age and duration of fever among participants with bacterial UTI and viral respiratory infection, with the former being of younger age and shorter duration of fever (Table 2). Considering the above-mentioned observation that neonates have lower sCAL concentration than infants, as well as weak correlation of sCAL with age (Table 3), the differences in sCAL among these two groups might be even higher, as probably are the differences in clinical appearance, prompting caregivers of children with UTI to visit an emergency department earlier and hence the lower duration of fever. Finally, 7 patients who initially presented as possible UTI due to the positive urinalysis, developed symptoms suggestive of respiratory viral infection while waiting for the results of urine culture, which came back negative. This situation is not uncommon in everyday clinical practice and therefore those participants were regarded as symptomatic respiratory viral infection with sterile pyuria and included in controls group. Nevertheless, similar sCAL concentrations were observed among these and other patients with proven viral respiratory infection and no significant differences were noticed if they are excluded from the analysis (data not shown).

In conclusion, compared to febrile patients with respiratory viral infection, sCal was significantly elevated in patients with bacterial UTI. Although it is clear that the diagnosis of UTI cannot be based on serum biomarkers and that urine culture remains the gold standard for diagnosis, our results suggest that sCal could have substantial added value in the early management of a child with fever and positive urinalysis and serve as an accurate biomarker in distinction between bacterial UTI and respiratory viral causes of febrile illness in children under 3 years of age.

## Data Availability Statement

The raw data supporting the conclusions of this article will be made available by the authors, without undue reservation.

## Ethics Statement

The studies involving human participants were reviewed and approved by University of Zagreb School of Medicine Institutional Ethics Committee and Sestre Milosrdnice University Hospital Institutional Ethics Committee. Written informed consent to participate in this study was provided by the participants' legal guardian/next of kin.

## Author Contributions

ML, LL, SA, and MH contributed to the study conception and design. Material preparation and data collection were performed by ML and LL. Formal analysis were performed by MMile, NN, MMilo, and SA. The resources were managed by MMile, NN, and MH. The first draft of the manuscript was written by ML and all authors commented on previous versions of the manuscript. SA was a supervisor. All authors read and approved the final manuscript.

## Conflict of Interest

The authors declare that the research was conducted in the absence of any commercial or financial relationships that could be construed as a potential conflict of interest.

## Publisher's Note

All claims expressed in this article are solely those of the authors and do not necessarily represent those of their affiliated organizations, or those of the publisher, the editors and the reviewers. Any product that may be evaluated in this article, or claim that may be made by its manufacturer, is not guaranteed or endorsed by the publisher.

## References

[1] Hammer HB Odegard S Fagerhol MK Landewe R van der Heijde D Uhlig T. Calprotectin (a major leucocyte protein) is strongly and independently correlated with joint inflammation and damage in rheumatoid arthritis. Ann Rheum Dis. (2007) 66:1093–7. 10.1136/ard.2006.064741 17234650 PMC1954700

[2] Vogl T Tenbrock K Ludwig S Leukert N Ehrhardt C van Zoelen MA. Mrp8 and Mrp14 are endogenous activators of Toll-like receptor 4, promoting lethal, endotoxin-induced shock. Nat Med. (2007) 13:1042–9. 10.1038/nm1638 17767165

[3] Romand X Bernardy C Nguyen MVC Courtier A Trocme C Clapasson M. Systemic calprotectin and chronic inflammatory rheumatic diseases. Joint Bone Spine. (2019) 86:691–8. 10.1016/j.jbspin.2019.01.003 30660804

[4] Lech M Guess J Duffner J Oyamada J Shimizu C Hoshino S. Circulating markers of inflammation persist in children and adults with giant aneurysms after Kawasaki disease. Circ Genom Precis Med. (2019) 12:e002433. 10.1161/CIRCGEN.118.002433 30844302

[5] Tyden H Lood C Gullstrand B Jonsen A Nived O Sturfelt G. Increased serum levels of S100A8/A9 and S100A12 are associated with cardiovascular disease in patients with inactive systemic lupus erythematosus. Rheumatology. (2013) 52:2048–55. 10.1093/rheumatology/ket263 23942785

[6] Lamot L Miler M Vukojevic R Vidovic M Lamot M Trutin I. The increased levels of fecal calprotectin in children with active enthesitis related arthritis and MRI signs of sacroiliitis: the results of a single center cross-sectional exploratory study in juvenile idiopathic arthritis patients. Front Med. (2021) 8:650619. 10.3389/fmed.2021.650619 33763437 PMC7982855

[7] Bartakova E Stefan M Stranikova A Pospisilova L Arientova S Beran O. Calprotectin and calgranulin C serum levels in bacterial sepsis. Diagn Microbiol Infect Dis. (2019) 93:219–26. 10.1016/j.diagmicrobio.2018.10.006 30420210

[8] Terrin G Passariello A Manguso F Salvia G Rapacciuolo L Messina F. Serum calprotectin: an antimicrobial peptide as a new marker for the diagnosis of sepsis in very low birth weight newborns. Clin Dev Immunol. (2011) 2011:291085. 10.1155/2011/291085 21765851 PMC3135082

[9] Decembrino L De Amici M Pozzi M De Silvestri A Stronati M. Serum calprotectin: a potential biomarker for neonatal sepsis. J Immunol Res. (2015) 2015:147973. 10.1155/2015/147973 26380313 PMC4563108

[10] Havelka A Sejersen K Venge P Pauksens K Larsson A. Calprotectin, a new biomarker for diagnosis of acute respiratory infections. Sci Rep. (2020) 10:4208. 10.1038/s41598-020-61094-z 32144345 PMC7060262

[11] Barbi E Marzuillo P Neri E Naviglio S Krauss BS. Fever in children: pearls and pitfalls. Children. (2017) 4:81. 10.3390/children4090081 28862659 PMC5615271

[12] Andersson KB Sletten K Berntzen HB Dale I Brandtzaeg P Jellum E. The leucocyte L1 protein: identity with the cystic fibrosis antigen and the calcium-binding MRP-8 and MRP-14 macrophage components. Scand J Immunol. (1988) 28:241–5. 10.1111/j.1365-3083.1988.tb02437.x 3413449

[13] Goyette J Geczy CL. Inflammation-associated S100 proteins: new mechanisms that regulate function. Amino Acids. (2011) 41:821–42. 10.1007/s00726-010-0528-0 20213444

[14] Pruenster M Vogl T Roth J Sperandio M. S100A8/A9: From basic science to clinical application. Pharmacol Ther. (2016) 167:120–31. 10.1016/j.pharmthera.2016.07.015 27492899

[15] Hessian PA Edgeworth J Hogg N. MRP-8 and MRP-14, two abundant Ca(2+)-binding proteins of neutrophils and monocytes. J Leukoc Biol. (1993) 53:197–204. 10.1002/jlb.53.2.197 8445331

[16] El Gazzar M. Immunobiology of S100A8 and S100A9 proteins and their role in acute inflammation and sepsis. Int J Immunol Immunother. (2015) 2:13. 10.23937/2378-3672/1410013

[17] Vogl T Gharibyan AL Morozova-Roche LA. Pro-inflammatory S100A8 and S100A9 proteins: self-assembly into multifunctional native and amyloid complexes. Int J Mol Sci. (2012) 13:2893–917. 10.3390/ijms13032893 22489132 PMC3317694

[18] Roberts KB. Revised AAP Guideline on UTI in Febrile Infants and Young Children. Am Fam Physician. (2012) 86:940–6. 23157147

[19] National Institute for Health and Care Excellence: Guidelines. Urinary Tract Infection in Under 16s: Diagnosis and Management. London: National Institute for Health and Care Excellence (NICE) Copyright © NICE 2020 (2018). 31971701

[20] Subcommittee Subcommittee on Urinary Tract Infection SCoQI Management Roberts KB. Urinary tract infection: clinical practice guideline for the diagnosis and management of the initial UTI in febrile infants and children 2 to 24 months. Pediatrics. (2011) 128:595–610. 10.1542/peds.2011-1330 21873693

[21] Bülbül A Bülbül L Zübarioglu U Ocak S Uslu S. The comparison of the management models for identifying the risk of serious bacterial infection in newborn infants with a newly developed scale. J Acad Res Med. (2020) 10:70–4. 10.4274/jarem.galenos.2018.2577

[22] Goldstein B Giroir B Randolph A International International Consensus Conference on Pediatric Sepsis. International pediatric sepsis consensus conference: definitions for sepsis and organ dysfunction in pediatrics. Pediatr Crit Care Med. (2005) 6:2–8. 10.1097/01.PCC.0000149131.72248.E6 15636651

[23] Shaikh N Morone NE Bost JE Farrell MH. Prevalence of urinary tract infection in childhood: a meta-analysis. Pediatr Infect Dis J. (2008) 27:302–8. 10.1097/INF.0b013e31815e4122 18316994

[24] Ruf BR Knuf M. The burden of seasonal and pandemic influenza in infants and children. Eur J Pediatr. (2014) 173:265–76. 10.1007/s00431-013-2023-6 23661234 PMC3930829

[25] Shi T McAllister DA O'Brien KL Simoes EAF Madhi SA Gessner BD. Global, regional, and national disease burden estimates of acute lower respiratory infections due to respiratory syncytial virus in young children in 2015: a systematic review and modelling study. Lancet. (2017) 390:946–58. 10.1016/S0140-6736(17)30938-8 28689664 PMC5592248

[26] Schlager TA. Urinary tract infections in infants and children. Microbiol Spectr. (2016) 4. 10.1128/microbiolspec.UTI-0022-2016 28087926

[27] Koufadaki AM Karavanaki KA Soldatou A Tsentidis C Sourani MP Sdogou T. Clinical and laboratory indices of severe renal lesions in children with febrile urinary tract infection. Acta Paediatr. (2014) 103:e404–9. 10.1111/apa.12706 24862642

[28] Shaikh N Borrell JL Evron J Leeflang MM. Procalcitonin, C-reactive protein, and erythrocyte sedimentation rate for the diagnosis of acute pyelonephritis in children. Cochrane Database Syst Rev. (2015) 1:CD009185. 10.1002/14651858.CD009185.pub2 25603480 PMC7104675

[29] Gelal A Gumustekin M Arici MA Gidener S. Rational pharmacotherapy training for fourth-year medical students. Indian J Pharmacol. (2013) 45:4–8. 10.4103/0253-7613.106426 23543821 PMC3608292

[30] Shaikh N Hoberman A Hum SW Alberty A Muniz G Kurs-Lasky M. Development and validation of a calculator for estimating the probability of urinary tract infection in young febrile children. JAMA Pediatr. (2018) 172:550–6. 10.1001/jamapediatrics.2018.0217 29710324 PMC6137527

[31] Sander J Fagerhol MK Bakken JS Dale I. Plasma levels of the leucocyte L1 protein in febrile conditions: relation to aetiology, number of leucocytes in blood, blood sedimentation reaction and C-reactive protein. Scand J Clin Lab Invest. (1984) 44:357–62. 10.3109/00365518409083820 6463565

[32] Castelli GP Pognani C Meisner M Stuani A Bellomi D Sgarbi L. Procalcitonin and C-reactive protein during systemic inflammatory response syndrome, sepsis and organ dysfunction. Crit Care. (2004) 8:R234–42. 10.1186/cc2877 15312223 PMC522844

